# Taurine is absent from amino components in fruits of *Opuntia ficus-indica*

**DOI:** 10.1186/2193-1801-3-663

**Published:** 2014-11-06

**Authors:** Hatem Salama Mohamed Ali, Abdulrahman Saleh Al-Khalifa, Hans Brückner

**Affiliations:** Department of Food Science and Nutrition, College of Food Science and Agriculture, King Saud University, P.O. Box 2460, Riyadh, 11451 Kingdom of Saudi Arabia; Department of Food Sciences, Research Center for BioSystems, Land Use and Nutrition (IFZ), Institute of Nutritional Science, Justus Liebig-University of Giessen, Heinrich-Buff-Ring 26-32, 35392 Giessen, Germany

**Keywords:** *Opuntia ficus-indica* (Cactaceae), Prickly pears, Taurine (2-aminoethanesulfonic acid), Amino acid analysis, Automated ion-exchange chromatography

## Abstract

Juices of edible fruits from *Opuntia ficus-indica* (L.) Miller, commonly named prickly pears or Indian figs, were analysed for amino acids using an automated amino acid analyser run in the high-resolution physiological mode. Emphasis was put on the detection of free taurine (Tau), but Tau could be detected neither in different cultivars of prickly pears from Italy, South Africa and the Near East nor in commercially available prickly pear juices from the market.

## Introduction

Taurine (2-aminoethanesulfonic acid) is among the most abundant amino compounds (second to the amino acid glutamine) in physiological fluids and tissues of the human body and is considered a conditionally essential amino-like compound. Consequently, there are a great number of reports on the physiological and clinical relevance of Tau [see Schaffer et al. (2014) for review].

Nutritional Tau has to be taken up in foodstuffs. Major natural sources of Tau are meat, milk and dairy products, as well as seafood. For infants, who cannot synthesise Tau, the dominant source is breast milk.

In food plants, however, Tau is rather scarce, and fruits and vegetables are not considered a source of Tau above sporadic trace concentrations [see Huxtable (1992) for a critical review]. Consequently, physiological fluids of strict vegetarians are low in Tau content, and consumption of food supplements containing this amino component is recommended.

Owing to the many reports dealing with the increase in physical and cognitive performance, drinks containing or even exceeding quantities of 4 g Tau/L are on the market and are widely consumed (Schaffer et al. 2014). The enormous quantities of Tau required are chemically synthesised on a large scale. Some individuals, however, reject uptake of ‘synthetic’ Tau.

Therefore, reports by Stintzing et al. (1999) and Tesorjere et al. (2005) on the occurrence of large (324–572 mg/L) or moderate (8.0–11.2 mg/100 g) quantities of Tau in the juice or pulp, respectively, of edible fruits of *Opuntia ficus-indica* (L.) Miller attracted attention.

The cactus plant is grown in favourable arid and semi-arid zones of all continents by rural communities, as well as in backyards and cultured orchards. Since fruits are marketed and exported on a large scale, prickly pears would represent the most abundant and easily accessible natural plant source of Tau.

Previously, we analysed edible date fruits from *Phoenix dactylifera* L. for proteinogenic and non-proteinogenic amino acids (AA) by automated ion-exchange chromatography (IEC) and realised that an intense peak eluting at the position of Tau was an artefact (Ali et al. 2014). Therefore, we analysed juices prepared from fruits of various cultivars of *Opuntia ficus-indica* using IEC; however, no Tau could be detected.

## Materials and methods

Intact and ripe fruits of *O. ficus-indica* from Italy (Sicily), South Africa and the Middle East with white, orange or red pulp were obtained from a local fruit markets. The surfaces of fruits were washed with distilled water, and then fruits were cut with a sterilised knife, and the pulps were strained through a metal mesh in order to remove the seeds. The resulting juices were heated at ~90°C for 30 min, then centrifuged at 16.000 x *g* for 10 min and then passed through a 0.25 μm filter (Sartorius). For analysis, the resulting supernatants were diluted 1:3 (v/v) with the common pH 2.2 buffer of the amino acid analyser and aliquots were injected using the 20-μL loop of the instrument. In addition, two batches of a commercially available juice from *O. ficus-indica* was analysed after dilution 1:4 (v/v) with the buffer. According to the manufacturer’s declaration, to these juices (purity 97%), small amounts of lemon juice of ‘organic’ origin were added.

**Figure 1:**
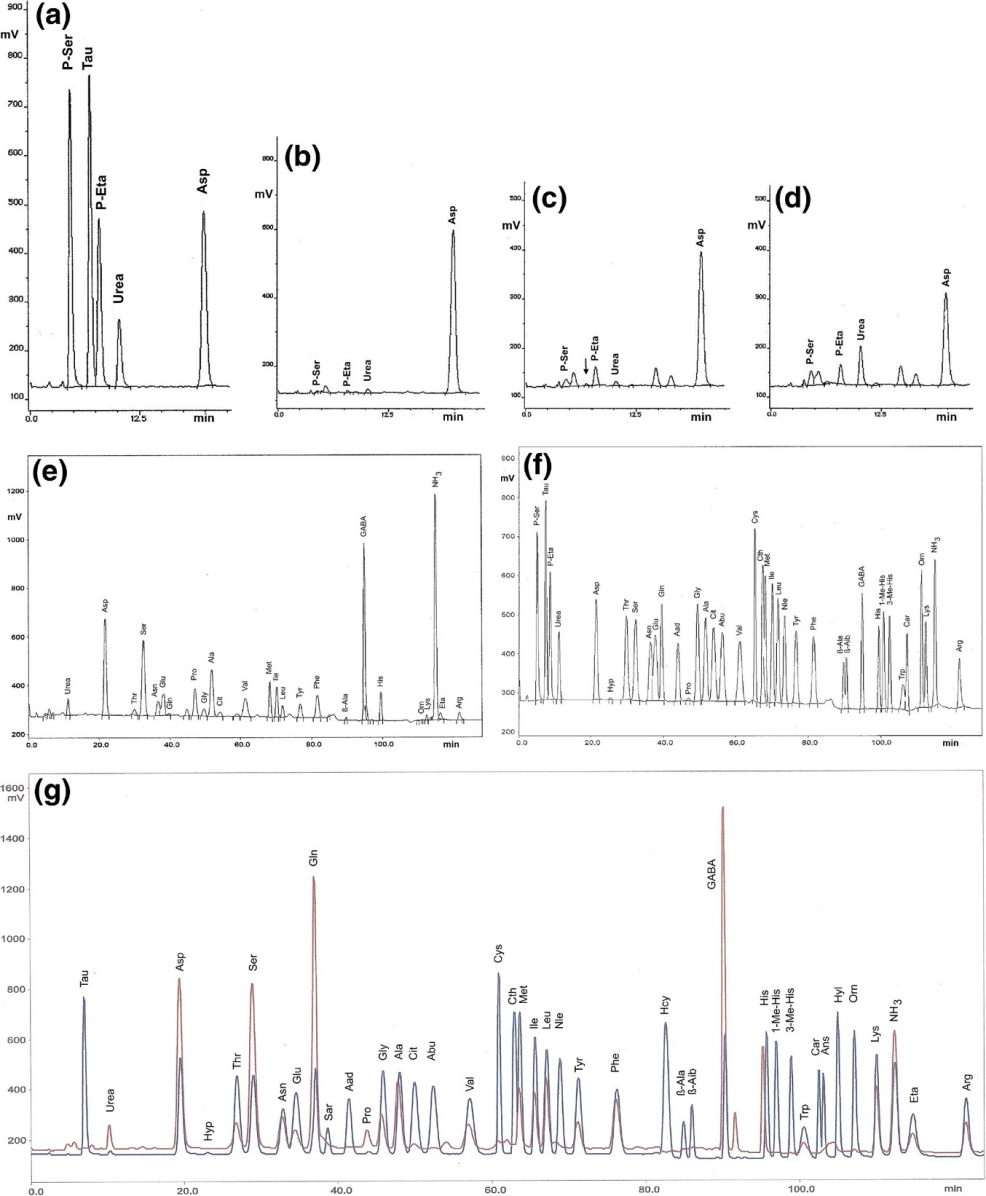
**Sections of chromatograms resulting from IEC of (a) a physiological standard showing compounds eluting at positions P-Ser, Tau, P-Eta, urea, and Asp, and of juices prepared from (b) white), (c) orange (for arrow see text), and (d) red pulp of** ***Opuntia ficus-indica*** **from Sicily, (e) a complete chromatogram of a commercial juice made from** ***O. ficus-indica*** **in comparison to (f) the physiological standard (BENSON) used for the assignment of peaks in (b) - (e), and (g) IEC of a juice prepared from the orange-yellow pulp of a South African prickly pear (upper trace) superimposed by a subsequently recorded chromatogram of a supplemented physiological standard from SIGMA-ALDRICH (lower trace).** Note that the chemical nature of compounds eluting at positions assigned as P-Ser, Tau, P-Eta and urea by the instrument, as well as of non-proteinogenic amino acids in **(e)** and **(g)** have not been confirmed by us by independent analytical methods. For acronyms of special amino components in standards **(f)** and **(g)** see list of abbreviations.

The analytical instrument used was a Biotronik LC 3000 automated amino acid analyser (Eppendorf-Biotronik, Hamburg, Germany) run in the physiological mode. Amino compounds eluted were derivatised with ninhydrin and photometrically measured at 570 and 440 nm (for quantitation of Pro). Quantification was performed by using external calibration and a physiological standard from BENSON Inc. (via BIOTRONIK) (see Figure 1f), and physiological standard A9906 from SIGMA-ALDRICH, St. Louis, MO, USA (see Figure 1g, lower trace). The SIGMA-ALDRICH standard was supplemented with Gln and Asn but did not contain P-Ser and P-Eta; the BENSON standard contained P-Ser and P-Eta but was lacking Eta. For full details of the instrument and chromatographic conditions, see Ali et al. (2014). Assignment of amino components was made by superimposing chromatograms of analytes and standards in subsequent runs; for an example see Figure 1g.

## Results and discussion

We analysed juices prepared from the pulp of fruits of cultivars of *O. ficus-indica* from the market and commercially available juices by employing an amino acid analyser run in the so-called physiological mode. Selected chromatograms of fruits distinguished by colour and country of origin, and of a commercial cactus pear juice are presented in Figure 1. In the standards used for calibration, the acidic Tau elutes at the beginning of the chromatogram and is well separated from P-Ser and P-Eta, as shown in the standard displayed in Figure 1a. In none of the juices obtained from the prickly pear cultivars Tau was detectable. Only in fruit juice no. 2, traces of a compound eluting close to the position of Tau could be detected (indicated by arrow in Figure 1c), but its chemical nature was not confirmed. However, if calculated for Tau, only 2.5 μmol Tau/L (corresponding to 0.26 mg Tau/L) would be present in this juice.

Notably, Askar and El-Samahy (1981) analysed Egyptian prickly pear fruits from *O. ficus-indica* by automated IEC using a Biotronik LC 6000 analyser. No Tau or components eluting before Asp are assigned in the AA elution profile shown.

Quantities of all amino acids and Eta detected in the prickly pear juices and assigned by comparison of retention times with the standards are compiled in Table 1. Besides common proteinogenic AAs and abundant GABA, non-proteinogenic Cit, Orn and Eta could be detected. In most juices relatively large amounts of urea were detected but its nature was not confirmed. Quantities and assignments of amino acids determined by IEC (with the exception of Tau) are in principal agreement with data resulting from HPLC analysis using OPA/thiol and gas chromatography – mass spectrometry (Stintzing et al. 1999, Kugler et al. 2006).

**Table 1:** **Quantities of amino compounds in μmol/L in juices made from the pulp of edible fruits of prickly pears (** ***Opuntia ficus-indica*** **) determined by automated ion-exchange chromatography**

	***1***	***2***	***3***	***4***	***5***	***6***	***7***	***8***	***9***	***10***	***11***	***12***	***13***	***14***	***Range***
Tau	ND	ND	ND	ND	ND	ND	ND	ND	ND	ND	ND	ND	ND	ND	ND
Urea	559.0	423.2	4702.2	16069.6	9520.3	9718.7	5230.7	4963.7	11057.2	2925.3	1293.8	9086.6	3895.6	4915.0	432 - 16070
Asp	808.9	460.0	330.1	1095.5	1295.0	730.6	686.8	789.4	849.0	883.2	830.3	857.2	1113.1	1089.7	330 - 12 95
Thr	97.2	47.4	27.0	189.5	125.9	169.7	150.5	141.3	137.3	171.4	134.1	54.8	122.0	121.8	27 - 190
Ser	868.8	475.1	323.7	1421.0	1150.0	973.4	1641.5	1740.1	174.3	927.7	752.7	894.2	1107.2	1101.4	174 - 1740
Asn	142.5	61.4	52.5	374.6	309.1	294.9	351.1	313.4	345.0	314.2	261.5	267.5	235.0	222.2	53 - 374
Glu	113.4	215.3	153.8	55.6	ND	96.0	31.3	41.7	54.9	127.3	140.4	235.5	353.3	386.7	ND - 387
Gln	2746.6	3185.8	1578.8	1068.0	1086.2	NQ	2346.6	1920.9	1896.1	1427.2	1422.2	294.6	25.3	NQ	25 - 3186
Pro	4784.8	4542.2	7299.7	7934.3	5809.4	7267.6	6053.3	8807.2	11161.0	3004.9	2263.9	4544.5	8443.3	8549.0	2050 - 11161
Gly	102.0	45.6	87.9	253.6	191.4	256.1	323.7	271.6	334.3	179.3	141.5	48.1	107.9	103.3	48 - 334
Ala	474.2	220.2	361.2	316.5	255.6	269.0	399.2	173.3	239.1	356.8	322.2	224.6	666.0	676.2	173 - 676
Cit	ND	18.2	39.1	283.2	110.35	79.5	52.2	55.6	145.3	21.7	16.7	62.2	64.5	80.3	ND - 283
Val	225.8	118.2	65.5	385.6	315.0	356.0	289.4	278.1	338.5	193.2	163.4	113.9	361.1	357.6	66 - 386
Cys	ND	ND	5.9	6.6	4.1	9.4	11.2	5.1	10.2	28.2	22.0	3.6	ND	ND	ND - 28
Met	120.3	43.8	28.8	232.4	201.9	210.0	232.6	240.1	186.3	189.1	159.0	187.0	312.9	311.5	29 - 313
Ile	167.8	98.1	54.8	426.9	363.0	499.8	207.4	218.9	252.9	218.2	187.4	79.7	298.0	306.5	55 - 500
Leu	237.6	142.8	86.0	359.6	261.7	346.4	272.6	233.4	240.3	317.0	259.1	45.4	128.5	131.7	45 - 360
Tyr	580.7	142.3	125.3	224.9	194.0	319.6	175.3	164.1	226.3	150.3	131.7	45.9	209.1	227.4	46 - 581
Phe	137.7	112.1	85.3	534.5	499.1	525.0	402.5	333.3	395.2	336.6	268.5	304.6	393.1	401.0	85 - 535
ß-Ala	ND	ND	ND	ND	ND	ND	33.0	26.6	42.3	ND	17.3	ND	62.7	71.6	ND - 72
GABA	1850.6	1063.8	1045.3	4220.4	3705.5	2556.7	3142.3	3445.6	3780.9	1238.2	1088.6	887.3	1862.4	1930.0	887 - 4220
His	600.5	810.1	145.6	668.6	511.0	769.7	531.5	563.4	675.6	357.9	302.7	330.6	304	ND	ND - 1456
Trp	65.1	55.6	93.4	151.7	ND	206.0	ND	ND	140.7	131.8	114.2	142.1	ND	73.0	ND - 152
Orn	6.0	9.2	3.9	15.6	3.9	ND	12.9	9.6	15.7	7.0	5.8	2.5	ND	ND	ND - 16
Lys	255.6	180.8	151.8	276.0	179.1	269.2	355.0	267.1	308.5	277.1	229.7	35.6	42.6	48.4	36 - 309
NH_3_	118.2	144.48	723.4	221.7	197.2	221.1	1217.7	543.5	922.4	552.4	490.3	476.4	2051.9	1331.8	118 - 2052
Eta	(++)	(++)	(++)	121.5	82.6	98.5	175.3	71.4	103.3	186.8	168.6	65.5	99.2	117.7	66 - 187
Arg	254.5	196.0	503.3	456.6	246.0	255.7	987.4	625.3	848.7	208.7	169.4	233.7	138.0	138.9	138 - 987

1 Green fruit, 2 orange fruit, 3 red fruit (from Sicily); 4-6 orange fruits (from Sicily); 7-9 orange fruits (from Silicy); 10, 11 two orange-red fruits (from the Republic of South Africa); 12, orange fruit (from the Middle East); 13, 14, two different batches of commercial juices from *Opuntia ficus-indica*; ND, not detected; NQ, not quantified; Eta (++), present in fruit juices 1–3 but not quantified; range, rounded values.

It is noteworthy in this context that IEC analyses of juices made from fresh plums by van Gorsel et al. (1992) did not provide evidence for the presence of Tau. In contrast, juices prepared from water extracts of dried plums (prunes) were reported to contain quantities of 100 - 155 mg Tau/L juice. Assignments by the authors were made by retention time. Analysis of commercial prune juice by us using high resolution IEC as described revealed that an intensive ninhydrin-positive peak elutes very close to Tau but definitely does not represent this compound (to be published by the authors). To sum up, there is currently no convincing data for the occurrence of Tau in nutritional fruit juices.

In agreement with our data, large quantities of GABA had been detected in prickly pear fruits (Askar and El-Samahy 1981, Kugler et al. 2006) and represent, together with Pro and Gln, the most abundant amino acids in prickly pear fruits. In plants, Gln serves as reservoir for excessive ammonium. After enzymic conversion into Glu, it serves as precursor of Pro and Arg. The latter is synthesised via intermediate Orn and Cit both of which have been detected in low amounts in the juices.

Gln is also the precursor of non-proteinogenic GABA in plants which is, like Pro, considered as response to biotic and abiotic stress (Bouché and Fromm 2004).

In most juices, relatively large amounts of urea were detected. In plants, urea mainly derives from catabolism of arginine by arginase (Witte 2011) but urea is usually not considered in fruit juice analyses.

From a nutritional point of view, the relatively large quantities of Gln, Arg, and Pro are of interest as these amino acids are considered as so-called functional amino acids, defined as key metabolic amino acids (Wu 2010).

## Conclusions

i) Tau is not a constituent of edible fruits of *Opuntia ficus-indica*.
ii) The relatively large or moderate amounts assigned as Tau in the literature in fruits of *O. ficus-indica* might result from confusion with GABA owing to the analytical method used (OPA/thiol) or, less likely, from special fruit treatment or use of manure.
iii) The most abundant amino acids in the fruits are Pro, Gln and GABA. Moderate amounts of free Eta could be detected in all juices, and low amounts of Cit and Orn in some juices. The presence of urea has to be confirmed further.

## Abbreviations

AA(s): Amino acid(s)
IEC: Ion-exchange chromatography
Tau: Taurine
P-Ser: Phosphorylserine
P-Eta: Phosphoethanolamine
Eta: 2-Aminoethanolamine
Hyp: Hydroxyproline
Sar: Sarcosine
Aad: α-Aminoadipic acid
Cit: Citrulline
Abu: α-Amino-*n*-butyric acid
Cth: Cystathionine
Nle: Norleucine (internal standard)
Hcy: Homocystine
ß-Ala: ß-Alanine
ß-Aib: ß-Amino*iso*butyric acid
GABA: γ-Aminobutyric acid
1-Me-His: 1-Methylhistidine
3-Me-His: 3-Methylhistidine
Car: Carnosine
Ans: Anserine
Hyl: 5-Hydroxylysine
Orn: Ornithine
Eta: Ethanolamine (2-Aminoethanol).
